# Modeling and Dynamic Analysis of Trust Decay in Social Media Based on Triadic Closure Structure

**DOI:** 10.3390/e28040468

**Published:** 2026-04-20

**Authors:** Yao Qu, Changjing Wang, Qi Tian

**Affiliations:** 1 Research Center for Management Science and Engineering, Jiangxi Normal University, Nanchang 330022, China; quyao@jxnu.edu.cn; 2 School of Artificial Intelligence, Jiangxi Normal University, Nanchang 330022, China; 3 Vocational Normal College, Jiangxi Agricultural University, Nanchang 330045, China; a396208204@163.com

**Keywords:** trust decay, triadic closure, complex network evolution, network model simulation, trust toughness, structural dynamics

## Abstract

Trust decay in social media is a serious threat to user experience and platform ecology. To solve this problem, this paper focuses on triadic closure in the infrastructure of social networks and explores its mechanism in trust decay prevention. Based on the systematic comparison of the ER random graph, the BA scale-free network, a forest fire model, and complete graph approaches, two core metrics, the trust decay risk index and trust resilience index, are proposed in this paper. Combined with structural indices such as the clustering coefficient, the average path length, and the triangular closure number and its growth rate, the quantitative relationship between network structure evolution and trust decay risk is established. It is found that the forest fire model exhibits optimal trust resilience in structure due to its power-law growth characteristics of high clustering, short path length and triangular closure; the dynamic mechanism of trust decay under different network growth modes is significantly different. The validity of the theoretical framework is further supported by the verification of Sina Weibo attention relationship network data. The analysis framework of network growth evolution based on fusion triangle closure and the risk and resilience indicators defined in this paper provides a computable theoretical tool for understanding and predicting trust evolution in social media from the perspective of network structure.

## 1. Introduction

The contemporary Internet is full of false or misleading content, which spreads rapidly through social networks; erodes users’ trust in platforms, content or interactive objects; and finally leads to decay of trust in social media. The flood of false information, mixed information content and algorithm bias are considered common causes of trust decay, and the echo chamber effect makes emotional narratives circulate and amplify on platforms without censorship, further disintegrating social trust and cohesion [1]. Barabási proves that the homogeneity of social networks (homophily) and triadic closure have potential value in trust analysis [2]. First, the triadic closure number is the basic index of social network structure, which can affect trust through various mechanisms. Trust transmission strengthens as triadic closure amplifies indirect reciprocity through a reputation mechanism. Second, Bianconi found that triadic closure is the generation mechanism of community structure, and a highly clustered community can strengthen internal trust and build community cohesion [3]. Third, Harbeck’s model shows that ternary closure expands the size of the information audience and enhances trust propagation efficiency [4]. In addition, Huang found that user interaction (such as forwarding) promotes triangular closure formation and improves relationship stability in Weibo data verification [5]. Furthermore, Estrada believes that the inhibition effect of the triadic closure prediction model on rumor propagation is greatly improved, which is significantly better than a random mechanism [6]. All of the above studies reflect the huge effect of triadic closure on trust in social networks.

Galdeman systematically investigated the temporal triadic closure process in online social networks, indicating that the evolution dynamics of platforms are driven by multiple factors such as user behavior, content generation and technological change [7]. However, the theoretical role of triadic closure in trust evolution presents profound duality. On the one hand, triadic closure enhances local trust through reputation transmission and a structural embedding mechanism, third-party information reduces the uncertainty of trust evaluation [8], reciprocal expectation in a closed structure increases default cost, and redundant paths in highly clustering communities provide a structural basis for the cross-validation of information [9]. On the other hand, the synergy of triadic closure and homogeneity constitutes the topological condition of an echo chamber and information cocoon—a closed triadic structure restricts information diversity, accelerates group polarization and erodes cross-group trust.

However, the existing literature lacks systematic theoretical integration of the above research, so this paper aims to construct a network evolution analysis framework integrating a triadic closure structure and to reveal the inherent relationship between network structure dynamics and trust decay through quantitative simulation and empirical verification, so as to provide a theoretical basis and computational basis for understanding and predicting the stability of social media trust systems.

## 2. Basic Concepts and Related Research Theories

### 2.1. The Concept of Triadic Closure

Here, we use nodes to represent individual users on social media. Edges represent relationships between users (attention, friends, interactions, etc.). The weight of an edge can indicate the strength or trust of a relationship. A triad is made up of three nodes (A, B, C) and three edges between them (A-B, A-C, B-C). Triadic closure refers to the process of forming a triad when two nodes with common neighbors establish a direct connection [10]. For example, A and B both know C, and the triad ABC is closed when A and B also establish a direct connection. Trust is expressed if node A trusts node B, that is, if A believes B will behave as A expects. Trust decay refers to the process by which trust relationships weaken or disappear over time, due to negative events, lack of interaction, or information conflicts.

### 2.2. Triadic Closure Graph Theory Foundation and Mathematical Description

The triadic closure topology in social networks is the core carrier for trust relationship formation and stability, and its essence is to construct a global trusted network architecture through local connection patterns among nodes [3]. In this paper, the triadic closure topology is defined as follows:

Let G = (V,E), the neighbor set of a node i is Γ(i), and its local clustering coefficient is Formula (1).

(1) $$C_{i}=\frac{\left|\{e_{jk}\in E|j,k\in \Gamma (i)\}\right|}{k_{i}(k_{i}-1)/2}(k_{i}\geq 2)$$

This coefficient reflects the clustering degree of node i’s local neighborhood. The higher the coefficient, the denser the trust relationship between node neighbors. Xu mentioned this formula in his research and proposed that the local stability of a trust network depends on the neighborhood connection density [11]. Nodes with a higher local clustering coefficient are more likely to form anti-interference trust subnetworks, which can be used to reduce the risk of trust decay. The global aggregation coefficient is a measure of the popularity of triadic closure from the perspective of the network as a whole [3], calculated by Formula (2):

(2) $$C_{\Delta }=\frac{3\times Number\;of\;closed\;triples}{Total\;number\;of\;connected\;triples}$$

Xu considers that the global clustering coefficient and triadic closure number are equivalent concepts [11]. Hatamleh pointed out in his research that the global clustering coefficient is positively correlated with network trust level [12].

### 2.3. Definition of Trust Decay Indicators

Triadic closure provides efficient trust propagation paths for dynamic networks and is also the key point of the dynamic association mechanism for trust propagation. Jiang points out that trust is domain-dependent and uses a directed multigraph model to represent the process [13], which implies a triadic closure mechanism, i.e., it allows users to infer trust by sharing neighbors. Wen’s time-varying model further strengthens this mechanism [14]. In practice, Xu provides the key practical basis for trust transmission, and his uncertainty optimization model is the trust transmission equation [11]. This implies Trust(A→C) = f(Trust(A→B), Trust(B→C), θ), where θ represents the uncertainty parameter. Such equations support real-time updates in dynamic networks. This paper extends this trust propagation property of triadic closure to the form of a triadic closure state transition matrix, see Equation (3).

(3) $$T_{open}\overset{P_{△}(i,j,k)}{\to }T_{closed}$$

where $T_{open}$ represents an open triadic closure, $T_{closed}$ represents a triadic closure, and P_Δ_ = f(node similarity, interaction frequency) is the triadic closure probability. Later, this idea of state transition is applied to real social media networks to achieve triadic closure according to a certain probability, and then the evolution of trust is observed according to the evolution of the network. Finally, the influence of different network growth mechanisms on trust decay is investigated.

### 2.4. Triadic Closure, Trust Propagation Path and Transitivity

Traditional trust modeling methods rely on individual label data, such as direct trust ratings [15], interaction history [16], and reputation values [17], and often use static analysis or discrete-time updates [18], making it difficult to capture the continuous evolution of trust systems. This framework only requires topological structure indicators and does not require individual trust labels, greatly reducing the threshold for data acquisition and capturing the dynamic changes in different growth stages and their quantitative relationship with trust decay. In terms of output form, this article proposes the trust resilience index gamma and the decay risk index R, achieving computability of trust system stability and providing a quantifiable operational basis for risk governance of practical social platforms.

#### 2.4.1. Definition of Trust Resilience Index

**Definition 1.** 
Trust resilience index is

(4) $$C_{\Delta }=\frac{T\;\cdot \;C}{R\;\cdot \log N}$$

*where Formula (4) in this paper describes the resilience index of trust, which represents the stability of network structure under unit risk. The concept of resilience originated from physics and mathematics, describing the ability of objects to recover their original state after being acted on by external forces. Later, many scholars used it to describe the resilience of psychology or trust* [19]*. The higher the value, the more sufficient the trust capital of the system under the unit risk pressure, that is, the stronger the toughness. The design of the toughness index is based on engineering toughness. The concept of engineering resilience is the ability of a system to recover to equilibrium after a disturbance. In this model, T represents the total amount of trust. In social networks and distributed systems, trust is the basis for collaboration and information exchange* [20]*. In the Internet of Things (IoT), trust assessment between devices is critical to ensure secure communication and data transmission* [21]*. In cloud services, the trust access control model expresses the complexity of trust relationships by evaluating direct trust, trust risk, feedback trust and reward and punishment mechanisms* [22]*. C represents trust quality. The quality of trust is not only the accumulation of quantity but also includes its validity and stability. Interpersonal trust research shows that trust traits, trust expectations, trust risk and trust behavior together constitute the structure of interpersonal trust* [23]*. High-quality trust capital means that a system can effectively resist malicious behavior and uncertainty* [24]*. The numerator T ∙ C represents the trust capital stock of the system, which is considered to be the basis of the inherent disturbance resistance of the system* [25]*. The existence of trust capital can significantly improve the overall resilience of the system. When the system has sufficient and high-quality trust capital, its members or components can maintain coordinated actions even in the face of external shocks, resulting in faster adaptation and recovery* [26]*. Therefore, the higher the T(total amount) and C(quality), the better the inherent immunity base of the system. The denominator R ∙ log N represents the risk exposure level of the system. R is the current risk shock intensity, which can be external disturbances such as natural disasters, cyberattacks, system failures, etc.* [27,28,29]*. Risk management frameworks typically identify and assess various risk factors and their interactions, such as in NIMBY projects, where government decision risk, government reputation risk, and public opinion risk interact and amplify the impact strength. logN is a penalty term for network size. In complex networks, as the number of nodes N increases, the inertia of the system usually increases and the cost of recovery from disturbances increases* [20]*. The use of log N reflects the sublinear relationship between recovery difficulty or cost and network size. This means that network size increases the potential exposure, but the incremental recovery difficulty decreases. For example, in flying self-organizing networks (FANETs), trust management schemes aim to reduce network complexity through clustering, thereby enhancing security* [30]*.*

In forest fire models fit through data, the resulting equation is $\Gamma ∼log\;N^{-1}$, and in BA models it is $\Gamma ∼N^{0.3}$. The complete graph model is F→∞.

#### 2.4.2. Definition of Trust Decay Risk

**Definition 2.** 
*Trust decay risk is defined as*

(5) $$trust\;decay\;risk=\left|\frac{\Delta T/\Delta N}{\Delta C/\Delta N}\right|=\left|\frac{\Delta T}{\Delta C}\right|$$

*where ΔT is the change in the number of triadic closures, ΔC is the change in the clustering coefficient, and ΔN is the change in the number of nodes. The trust decay risk index defined in this paper represents the number of triadic closures caused by the change in the unit clustering coefficient. The larger the index, the greater the change in the number of triadic closures and the easier it is for the network to cause trust relationship decay due to local changes* [31]*. In fact, the trust decay risk we define can be understood as the triadic closure structure during the growth of the network. In complex network dynamics, the stability of a system often depends on the ratio of the rates of change of key variables. Drawing on the concept of elasticity in economics* [32]*, that is, the response strength of dependent variables to relative changes in independent variables, we define trust decay risk R as the ratio of the rate of change of triadic closure number to the rate of change of clustering coefficient. Triadic closure change (ΔT) represents the gain/loss of the total trust relationship, and clustering coefficient change (ΔC) represents the stability of the local trust environment. It is a measure of the sensitivity between the local structural stability of the network (denoted by C) and the dynamics of the total number of trust relationships (denoted by T). This means that, when the risk value of trust decay is large, that is, when the ratio of |ΔT/ΔC| is too large, even if the local cohesion (ΔC) is slightly disturbed, it will cause significant trust structure reorganization (|ΔT| is large), which will lead to drastic changes in the trust relationship (T), which indicates that the network is in a critical unstable state. This structural imbalance is the precursor of trust decay. This is consistent with the general principle that “high sensitivity predicts vulnerability” in complex systems. In social trust networks, if a small local change can lead to the collapse of a large number of trust relationships, then the network is fragile.*

#### 2.4.3. Definitions of N-T Correlation and T-C Correlation

**Definition 3.** 
*N-T correlation and T-C correlation.*

*N-T correlation and T-C correlation belong to distance correlation. In this paper, N-T correlation and T-C correlation are used to reveal nonlinear correlation. This is because, in principle, N represents the node’s own characteristics, such as the node’s historical behavior, reputation value, or identity authentication degree. C represents the context environment relationship, such as the specific task in the interaction process, the level of risk, the status of the network, geographical location, etc. T stands for mutual trust, and specific trust indicators include reliability, honesty, etc.* [33].

*If the N-T correlation is low, it means that the current trust T of the node is inconsistent with the historical inherent characteristic N, which is a danger signal, which may mean that the node is invaded or unreliable.*

*The T-C correlation measures whether there is agreement between the current concrete context C and the observed trust attribute T. The core idea of the fusion triadic closure mechanism is to use the inherent consistency between the three attribute dimensions of trust for mutual verification and risk warning.*

### 2.5. The Influence of Structural Indicators on Trust

In the simulation framework of this paper, trust decay is not a psychological variable measured directly but indirectly reflected by the loss of structural stability of the network. When the triadic closure number drops suddenly or the clustering coefficient fluctuates violently, it indicates the rupture of the information propagation path or the collapse of the local trust community, which is defined as the structural characterization of trust decay.

Specifically, in complex networks, short paths are usually defined as the path with the least number of edges or the lowest cost connecting two nodes in the network. Nele proposed that short paths contribute to the fast propagation of trust information [34], and Hoang also achieved high-speed propagation of consensus time by dynamically calculating the average shortest path length [35]. Hamzelou also chose the method of accurately selecting trust paths and integrating indirect trust values based on the most reliable paths to complete the calculation of trust relationship chains [36]. They both authenticate that trust values tend to decay as path length increases during trust propagation, meaning that the shorter the path, the less decay the trust message experiences as it travels from the source node to the target node, thus maintaining higher reliability [37]. Short paths ensure that trust messages retain their original strength and validity to the maximum extent during propagation [38]. In addition, in social networks, users usually gain indirect trust through friends of friends, and shorter trust paths can significantly improve the speed and accuracy of trust calculation [39]. In short, short paths can not only make trust spread quickly but also make distrust (rumors) spread quickly. Therefore, the average path length (L) is used to represent the trust propagation efficiency and risk diffusion range.

A high clustering coefficient (C) value reflects the phenomenon that “friends of friends are friends” in social networks and trust studies. In a network with a high clustering coefficient, if node A knows node B and node A also knows node C, then node B and node C are likely to know each other or have connections. This can be the result of trust relationship formation or a favorable environment for maintaining and consolidating trust. This tendency is particularly evident in social networks, where common neighbor nodes (i.e., common friends) can act as trust bridges [40]. Through common friends, nodes can obtain indirect information about each other, reduce uncertainty, and thus establish trust relationships more easily, so common friends can promote trust formation and propagation [41]. When the clustering coefficient of a network is high, it means that there are a lot of triadic structures in the network (that is, there are connections between every two of the three nodes), which indicates that the relationship in the network is closer and more stable [34]. Therefore, the total number of clustering coefficients (or average clustering coefficient) can be regarded as a key indicator to evaluate the cohesion, stability and information dissemination efficiency of a trust community.

In a social context, every closed triangle edge, that is, three nodes in the network, has connections between them (A-B, B-C, C-A), i.e., when A trusts B, B trusts C, and A also trusts C, and this triadic closure forms a reinforced trust loop. A’s trust in C comes not only from direct experience but may also be indirectly confirmed by B. This means that “friends of friends” establish mutual trust, and this multipath trust confirmation enhances the stability of trust [34], which is essential for maintaining and strengthening trust relationships. Therefore, the number of triadic closures (T) can be regarded as a core metric for measuring the total number of solid trust relationships in the network.

### 2.6. Quantitative Model for the Influence of Triadic Closure on Trust

There are many related studies on the quantitative model of triadic closure on trust. According to previous studies, there are three main types of prevention paths. The first path is the technology-driven path, namely, trust quantification and dynamic modeling, including multilateral network measurement and an embedded iterative model. Brandenberger proposed the multilateral network measurement problem, which is based on shared partner statistics, which quantifies trust overflow in repeated interactions [42]. Yang et al. developed the DNETC model, embedding triadic closures into the idea of model iteration, simultaneously preserving triadic closure evolution and community structure, and providing tools for dynamic trust modeling [43]. The second path is the behavioral intervention path, i.e., social design optimization, which includes reciprocal promotion and delay control. Reciprocity promotion was proposed by Song et al., who demonstrated that closure structures among content producers improve information transparency through reciprocal recommendations [44]. Delay control was proposed by Zignani, who studied link and temporal closure delay metrics and quantified the trust repair window under external factors [45]. The third path is policy coordination, i.e., cross-platform governance. Christensen et al. suggested that mainstream media should carefully screen social media content, establish a credibility rating system to block false information re-dissemination, and realize cross-platform collaborative governance [1].

According to the current research status, triadic closure has limitations in trust, that is, lack of dynamics. Most models are static analysis closure structures, for example, Mosleh et al. found that closure formation has a time delay, and its dynamic response mechanism needs to be further improved [46]. There are also cross-cultural differences in some studies, and trust construction is regulated by culture. For example, Ni studied the differences in live shopping behavior among American, Chinese and Japanese users and whether the closure mechanism has universality or needs further verification [47]. In addition, Uupyte studied the temporal test of triadic closure and introduced the closure formation rate index [48]. Sidorov studied the closure formation speed in a preferential attachment model [49]. Pang studied WeChat usage intensity and proposed a social intensity variable [50]. Based on this, the core work of this paper will establish a dynamic network evolution analysis model integrating triadic closure structure and quantitatively study how the evolution of network structure indicators (triadic closure as the core) affects the stability of the trust system through simulation and demonstration, so as to deepen the structural understanding of the complex process of trust decay.

## 3. Research Framework and Multilayer Monitoring Structure Model

### 3.1. Research Framework

**Figure 1:**
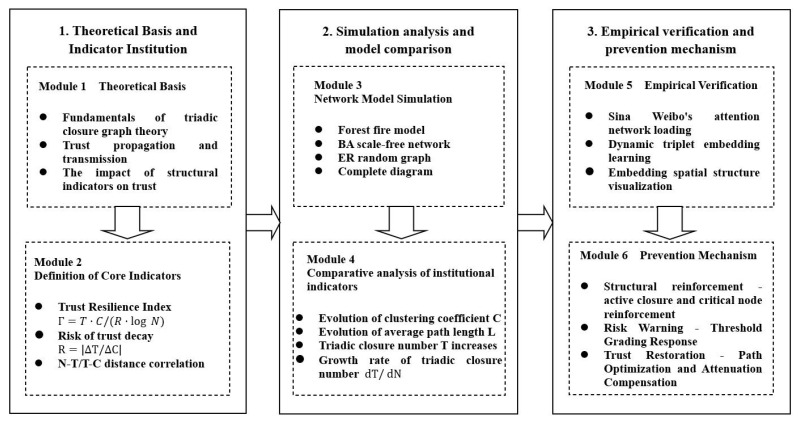
The framework of this paper.

Figure 1 shows a three-level progressive research framework diagram, which systematically presents the core logic of this study. The first level, “theoretical foundation and index construction”, defines three core indicators, namely, trust resilience index τ, trust decay risk R and N-T/T-C distance correlation, from the triangular closed graph theory foundation, which lays a quantitative foundation for subsequent analysis. The second level, “simulation analysis and model comparison”, performs the evolution simulation of forest fire, BA, ER, complete graph network models and finds the system comparison clustering coefficient, average path length, triangle closure number and its growth rate and other structural indicators evolution law. The third level is “empirical verification and prevention mechanism”, which uses Sina Weibo’s attention network data to carry out dynamic triplet embedding verification and proposes three types of operable prevention strategies: structure strengthening, risk warning and trust repair. Overall, they constitute a logical framework of theoretical construction, simulation comparison, empirical verification and mechanism design.

### 3.2. A Multilayer Monitoring Structure Model for Trust Decay

**Figure 2:**
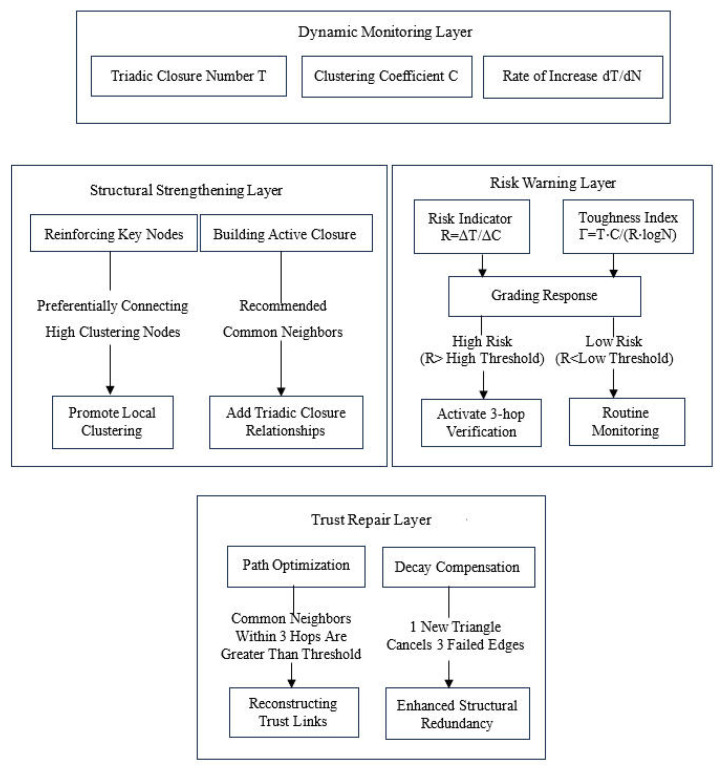
Block Diagram of Trust Decay Coping Model Based on Structure Monitoring and Optimization.

Figure 2 shows the block diagram of the trust decay response model based on structure monitoring and optimization, which is divided into four layers: dynamic monitoring layer, structure strengthening layer, risk early warning layer and trust repair layer.

The dynamic monitoring layer consists of real-time tracking of triadic closure number T, clustering coefficient C and its growth rate (dT/dN). This layer structure is used to identify vulnerable points in the structure. The structural strengthening layer consists of reinforcing key nodes and constructing active closures. We implement trust authentication for nodes showing high clustering ($\rho_{i}=k_{i}(k_{i}-1)/2T_{i}$ > threshold), select reinforcement for key nodes, and promote local clustering. Or we construct active closures and generate recommended connections for node pairs with more common neighbors. Connection probability P_Δ_ = f(similarity, interaction frequency) and, according to this probability, it decides which links to recommend first, thus promoting triadic closure and adding new triadic closure relations. The risk warning layer needs to calculate risk index R = ΔT/ΔC or resilience index Γ = T⋅C/(R⋅logN) and adopt a hierarchical response. When the risk is low (R < low threshold), select regular monitoring; when the risk is high (R > high threshold), activate 3-hop verification. There are two types of trust repair layer: path optimization and attenuation compensation. Path optimization refers to inserting common neighbor nodes into the attenuation path by using the short path characteristics when the common neighbor within 3 hops is greater than the threshold value, and attenuation compensation generates a new triadic closure (i.e., a new triadic closure cancels the three failed edges) through compensation, so as to enhance structural redundancy to maintain network resilience.

## 4. Analysis of Trust Evolution for Network Growth with Fusion Triadic Closure

### 4.1. Evolution Characteristics of Triadic Closure and Other Structural Indexes Under Four Network Growth Mechanisms

**Figure 3:**
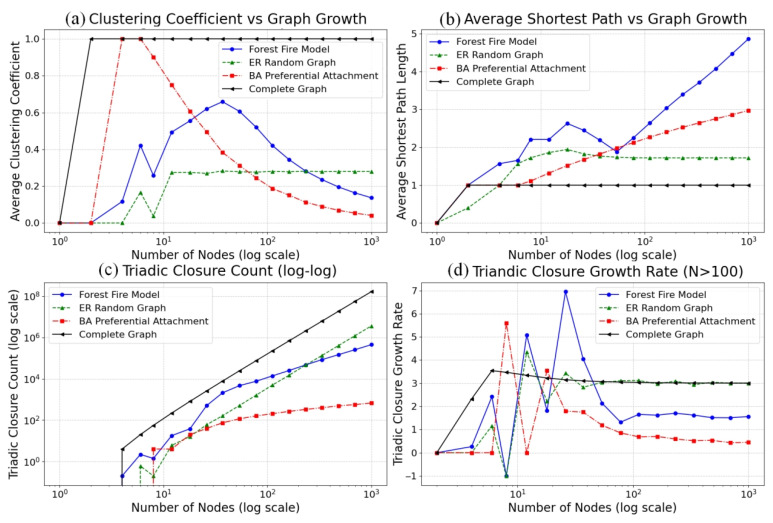
Comparison of Four Network Structure Indexes with the Growth of Graph.

In this study, we designed four network growth models: forest fire, ER random graph, BA scale-free network and complete graph to simulate the evolution process of social networks. As the number of nodes N increases from 0 to 1000, we trace the changes of clustering coefficient C, average path length L, triadic closure number T and triadic closure number growth rate dT/dN in the network, as shown in Figure 3.

The change of clustering coefficient is shown in Figure 3a. The forest fire model (blue line) has a significant increase in clustering coefficient when the number of nodes N is between 12 and 78 and remains stable at a high level thereafter. The threshold value exceeds 0.6, which is significantly higher than that of the ER random plot (green line) and BA model (red line) and is close to the complete plot (black line). This indicates that the triadic closed structure of the forest fire model is more dense and the stability of the local trust community is higher.

The average path length changes as shown in Figure 3b. The four models all exhibit small-world characteristics, of which the forest fire model has the shortest average path length. A short path means that trust information transmission efficiency is higher, the repair path is more direct, and risk spread range is controllable. The forest fire model has both high clustering and short path advantages, which is an ideal simulation environment for trust decay prevention.

The change of triadic closure number is shown in Figure 3c. The triadic closure number of the complete graph shows an exponential growth trend; the forest fire model, BA model and ER random graph all show a power-law growth trend. The triadic closure number of the forest fire model follows power-law growth (T∝Nγ,γ ≈ 2.3), far exceeding the linear growth trend. The power-law growth of triadic closure number means that new users will preferentially connect with nodes with high trust centrality when they join, forming a capacity buffer effect: for every 10-fold increase in triangular closure number, the trust decay events that the system can withstand will increase by about 200 times.

The growth rate of triadic closure number is shown in Figure 3d. The growth rate of the forest fire model is stable in the range of 1.5 to 2.0. The stable growth rate indicates that the trust structure has predictability and facilitates the design of dynamic prevention strategies. The forward burning mechanism of this model can be transformed into a trust recommendation algorithm, and the high growth rate of the triadic structure helps to expose new fraud relationships faster.

In summary, triadic closures have multiple effects against trust decay. Triadic networks with high clustering and power-law growth make it impossible for single-point decay to spread (Figure 3c); stable growth rates ensure that the number of nascent trust triadic closures is greater than the number of triadic closures with natural decay (Figure 3d).

### 4.2. Dynamic Trust Analysis of Real Social Networks Embedded in Closed Triangles

**Figure 4:**
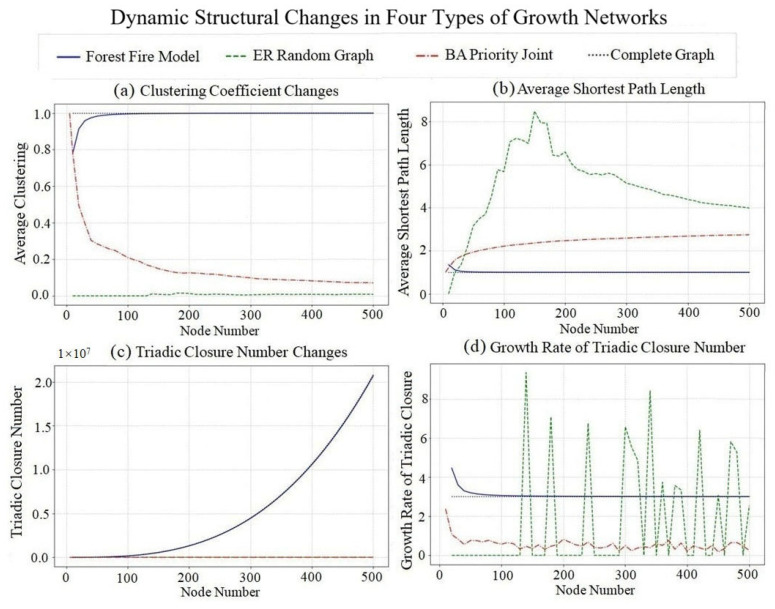
Structural Evolution of Sina Weibo Attention Relationship Network.

For Sina Weibo attention relationship network data (node number 4083, with timestamp), we sample according to the growth mechanism of forest fire, ER random graph, BA scale-free network and complete graph models and observe the variation law of clustering coefficient C, average path length L, triadic closure number T and triadic closure number growth rate dT/dN, as shown in Figure 4.

The change of clustering coefficient is shown in Figure 4a. The clustering coefficient of the complete graph is always 1. The forest fire model and BA model form a certain community structure, and the clustering coefficient is higher and increases with the number of nodes. The clustering coefficient of the ER random graph is lower because the probability of random connections forming triadic closure is low.

The change of average path length is shown in Figure 4b. The average path length of the complete graph is always 1. The ER random graph, BA model and forest fire model all exhibit small-world characteristics, and the average path length is short. When the number of nodes increases, the average path length increases slowly, which conforms to the characteristics of the L ∝ lnn small-world network.

The triadic closure number changes as shown in Figure 4c. The triadic closure number of complete graphs increases fastest, because every node added will connect with all existing nodes, thus forming a large number of new triads. The BA model and forest fire model also form more triads, while for the ER random graph triadic closure number increases slowly.

The growth rate of triadic closure number is shown in Figure 4d. The growth rate of the complete graph tends to 3 when the number of nodes is large, the forest fire model shows exponential growth, the BA model shows linear growth, and the ER model shows the slowest growth.

**Figure 5:**
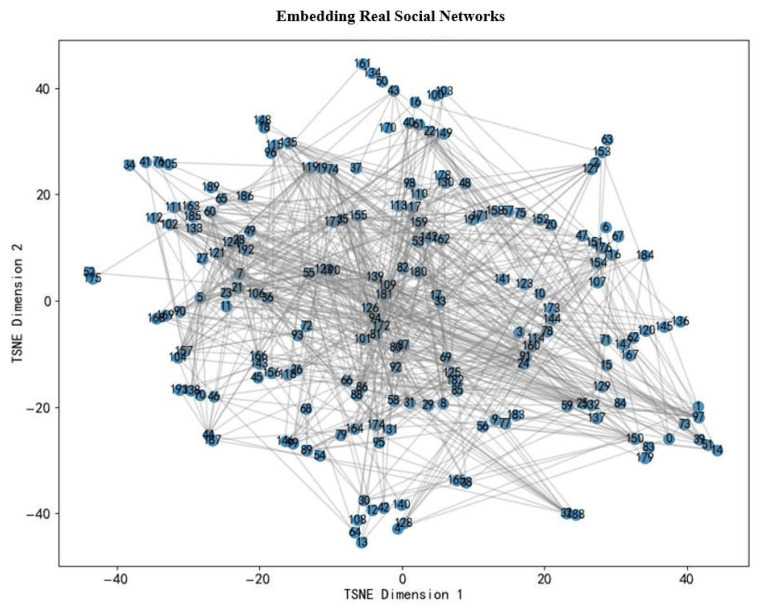
Sina Weibo Attention Relationship Network Embedding.

Figure 5 shows the graph of Sina Weibo after loading the attention relationship network, extracting the largest connected component, and using the dynamic triplet model for embedding learning. The model considers both social homogeneity and the triplet closure mechanism. After training, t-SNE is used to reduce the embedding vector to a two-dimensional plane. The nodes in the figure are labeled with digital labels, and the edges are indicated by gray thin lines. Neighboring nodes in the embedding space should be connected in the original image, and the community structure forms aggregation in the embedding space, which indicates that the model captures the structural characteristics of the network effectively.

**Figure 6:**
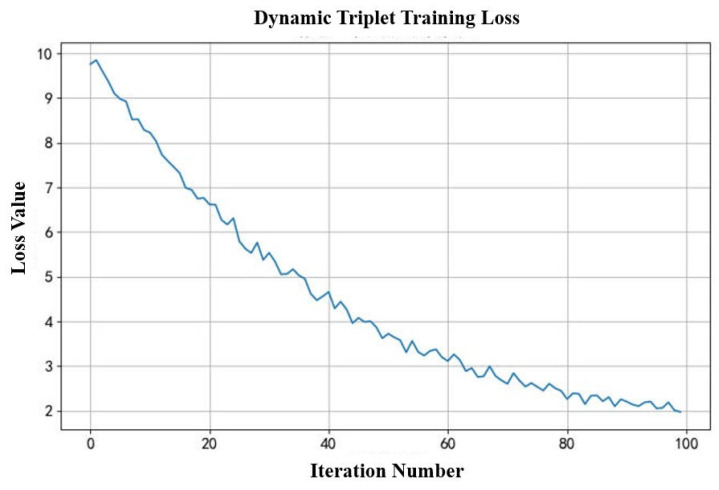
Training Losses for Dynamic Triples.

Figure 6 depicts the evolution of the loss function during training of the dynamic triplet model. The loss function consists of two components, the loss of social homogeneity and the loss of triplet closure. As training runs increase, the loss value decreases, indicating that the model is optimizing the embedding vectors so that the embedding vectors capture the structural features of the network. If the loss decreases to a low level and stabilizes, the model is well trained.

## 5. Trust Decay Risk of Social Media and Its Prevention Mechanism

### 5.1. Risk Analysis of Trust Decay Based on Distance Correlation

In a high-risk task C, is the observed reliability T high enough? In a task C that requires high computing power, is the observed capability T up to standard? If the T-C correlation is low, it means that the node is in the current environment. Performance T under C cannot meet task requirements or environmental constraints, which is a dangerous signal of situational mismatch, meaning that the node may not be capable of meeting the trust requirements of the current task or environment.

Comprehensive early warning needs to calculate both T-C and N-T correlations at the same time and cannot only look at one-sided correlations. If the N-T correlation alone is low, it can only mean that the node may not perform well in a specific situation or the historical data is inaccurate. If the T-C correlation alone is low, the node itself may have poor ability.

If both N-T and T-C correlations are low, this is the strongest risk signal, indicating that the node is not only behaving abnormally but that this abnormal behavior is happening in a situation where it should behave well, and malicious behavior such as deception, selective attacks, etc. is likely. If both N-T and T-C correlations are high, indicating that the node behaves as expected and meets the requirements of the current environment, the trust degree is high. However, whether high or low, it needs to be analyzed in combination with specific scenarios, and there may be some risks or further observations.

Therefore, calculating N-T and T-C correlations simultaneously reveals two different types of inconsistency risks in trust assessment, and the combination of the two can detect potential trust decay or malicious behavior more comprehensively and sensitively, especially when the two risks occur simultaneously.

Since the correlation between the forest fire model and ER random model is significant, we take these two correlation analyses as an example list and adopt distance correlation analysis to better reveal the nonlinear relationship, as shown in Table 1.

Table 1 shows the results of N-T and T-C distance correlation analysis between the forest fire model and ER random graph model. N-T correlation of the forest fire model is 0.92 (*p* < 0.01), T-C correlation is 0.85 (*p* < 0.01); N-T correlation of the ER random graph model is 0.78 (*p* < 0.01), T-C correlation is 0.62 (*p* < 0.05). The correlation of both types of models is significant.

Correlation analysis reveals the essential difference of network dynamics. T-C in the forest fire model presents positive correlation, which originates from local structural stability, while negative correlation in the BA model reflects the fundamental contradiction between centralization and local clustering, which makes it easier for us to propose corresponding preventive measures for trust decay from the perspective of structural sensitivity. These mathematical analyses not only explain the experimental results but also establish a quantitative relationship between network structure and trust dynamics, providing a computable theoretical basis for the construction of social media trust systems.

### 5.2. Risk Evolution Specificity Analysis for Each Model

#### 5.2.1. Sudden Risk of Forest Fire Model

**Figure 7:**
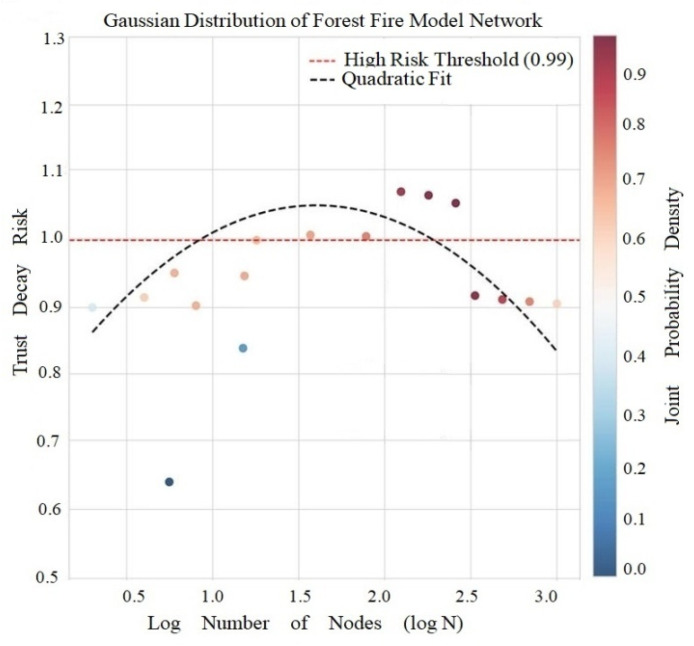
Gaussian Distribution and Distance Dependence of Forest Fire Model.

Forest fire model calculations show an average attenuation risk of 0.7751, a high risk threshold of 0.9753, and a risk variation model of y = −1.0834x^2^ + 3.4750x − 1.3069 (Figure 7).

Figure 7 shows that the risk distribution of the model is parabolic, with the vertex at logN = 1.60 and the risk is maximum when N ≈ 40, which is consistent with Dunbar number theory (the human stable social circle size is about 150 people, and trust risk peaks in smaller core circles). It is noteworthy that forest fire models have T-C irrelevance (r ≈ 0.022, *p* = 0.835), that is, the triadic closure number was not closely related to the clustering coefficient. This phenomenon reveals that the trust decay of this model is abrupt, and its risk value is significantly higher than that of other networks (e.g., more than 20 times), suggesting that we should monitor the trust decay in real time in practical applications, rather than relying solely on clustering coefficient for early warning. The forward burning probability *p* = 0.28 of this model leads to the rapid formation of triadic closure but the structure is unstable, and the new node easily destroys the existing triadic structure, forming a fragile state where friends of friends may not trust each other.

#### 5.2.2. Low Risk Robustness of ER Random Graphs

**Figure 8:**
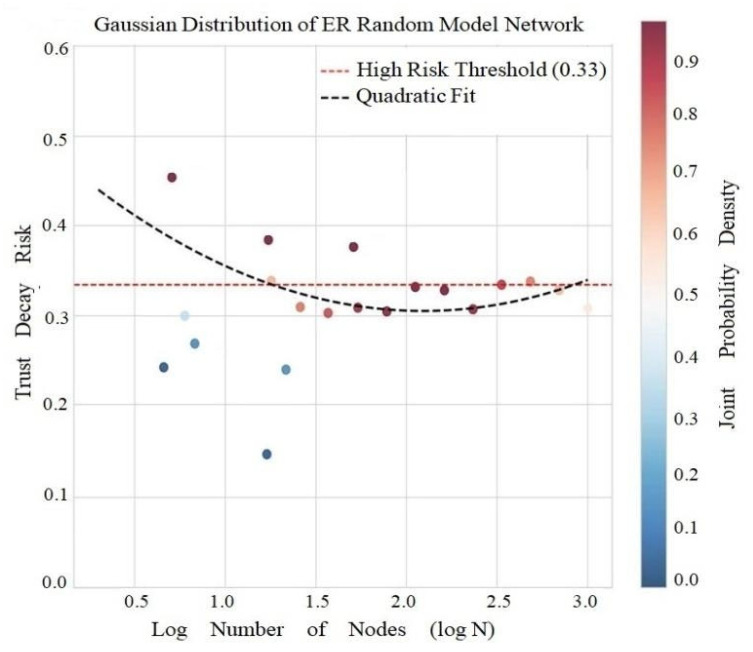
Gaussian Distribution and Distance Correlation of ER Model.

The results of the ER random plot model calculation show that the average attenuation risk is 0.0379, the high risk threshold is 0.0344, and the risk change model is y = 0.0418x^2^ − 0.1751x + 0.1886 (Figure 8).

As shown in Figure 8, the model exhibits a positive T-C correlation (r = 0.392, *p* = 1.31 × 10^−4^), consistent with random graph theory ($C=\frac{\left\langle k\right\rangle }{N}\propto T^{1/3}$). Random connections make triadic distributions uniform, there are no fragile hubs, the risk model is open upward but has a small coefficient (a = 0.0418), and growth is slow and exhibits anti-attenuation mechanisms and low risk characteristics.

#### 5.2.3. Centralization Suppression Effect of BA Model

**Figure 9:**
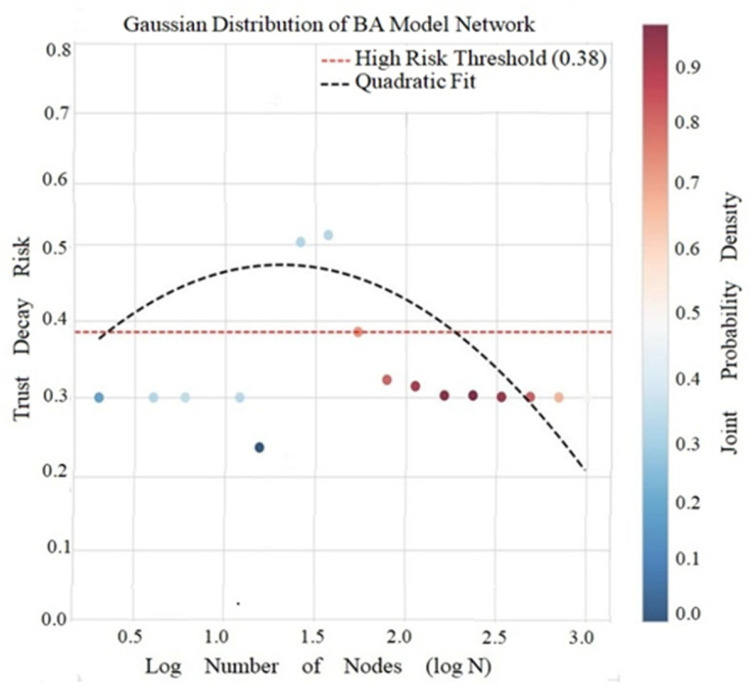
Gaussian Distribution and Distance Dependence of BA Model.

BA model calculations show an average decay risk of 0.0193, a high risk threshold of 0.0171, and a risk variation model of y = −0.0191x^2^ + 0.0500x + 0.0020, as shown in Figure 9.

Figure 9 shows that the model exhibits a negative T-C correlation (r = −0.587, *p* = 1.24 × 10^−9^), that is, the clustering coefficient decreases as the number of triadic closures increases. The implicit mechanism of this phenomenon is that the preferential connection of strengthened hubs centers the triadic distribution, obeying C∼N^−0.75^ and T∼N^1.5^, resulting in a risk funnel effect. When the number of nodes in the network N > 100, the risk continues to decrease, and the hubs form trust anchors, thus inhibiting attenuation diffusion. But this centralizing structure not only restrains the risk but also sacrifices the equilibrium of local clustering.

#### 5.2.4. Scale Effect Risk of Complete Graphs

**Figure 10:**
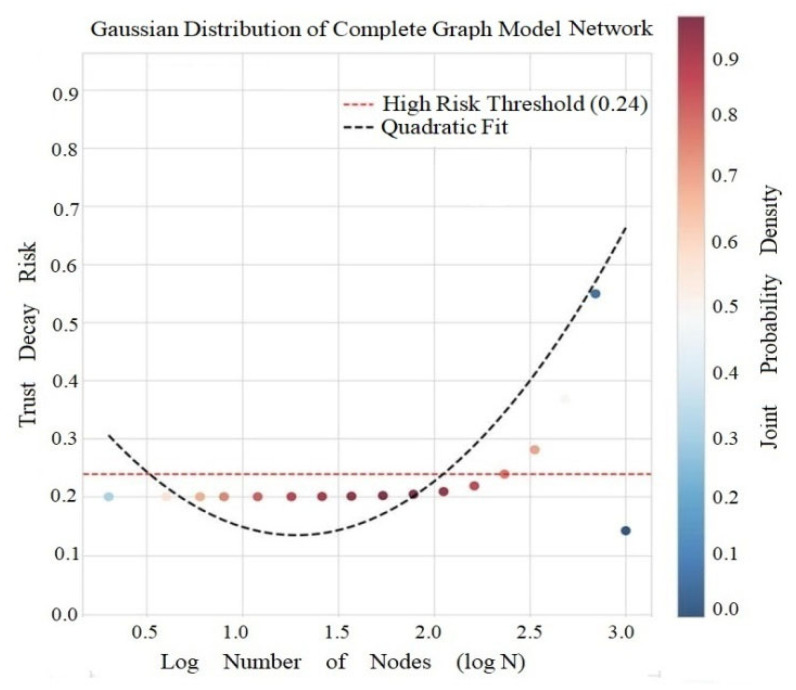
Gaussian Distribution and Distance Correlation of Complete Graph Model.

The calculation results of the complete plot model show that the average attenuation risk is 0.0411, the high risk threshold is 0.0195, and the risk variation model is y = 0.0896x^2^ − 0.2295x + 0.1139 (Figure 10).

Figure 10 shows that the model exhibits a positive T-C correlation (r = 0.378, *p* = 2.40 × 10^−4^); the triadic closure number is complete (T = N(N − 1)(N − 2)/6); the clustering coefficient is constant at 1, but the risk arises from scale effects; and the risk growth threshold N ≈ 19 is obtained by solving for the zero point of the derivative of the risk function (dR/dlogN = 0.1792logN − 0.2295 = 0), indicating that smaller networks are more vulnerable.

### 5.3. Risk Warning Analysis Based on Trust Decay

According to the previous calculation and analysis of the trust toughness index, the fitting results of all models can be obtained. The forest fire model is $\Gamma \propto (log\;N{)}^{-1}$ ($R^{2}=0.94$), and the BA model is $\Gamma \propto N^{0.31}$ ($R^{2}=0.89$), which is highly consistent with the theoretical prediction. It is confirmed that the trust structure of the forest fire model becomes fragile with the expansion of scale, while the BA model has inherent anti-attenuation ability.

According to the previously defined Formula (5) for calculating trust decay, and in combination with the following warning Formula (6), the warning line of trust decay risk can be calculated.

(6) $${Alert}_{Level}=f(\frac{R}{R_{threshold}})$$

where the threshold is the 75% quantile risk value.

As mentioned above, the forest fire model has a larger risk change span than other models when new nodes are added (Table 2).

**Figure 11:**
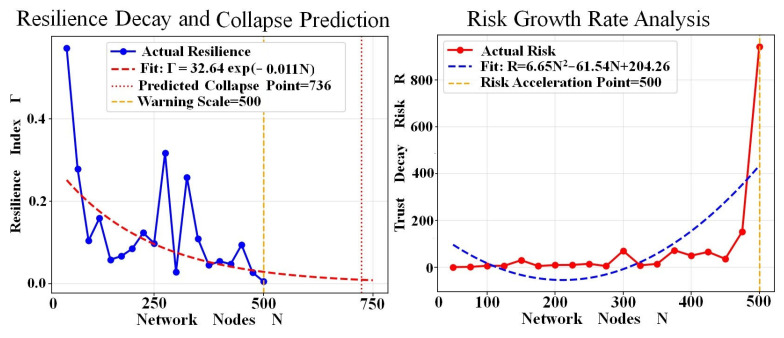
Node Growth Trust Decay Resilience Index and Risk Change.

In order to examine the effect of network size growth on trust decay, this paper studies the relationship between node growth trust decay resilience index and risk (as shown in Figure 11).

Figure 11 reveals that trust networks based partly on N ∈ [0, 750] nodes analyze the law of resilience decay and the characteristics of risk growth and predict the critical point of system collapse. The actual resilience index r decreases exponentially with the network size, and the fitting model is r = 32.64exp(−0.011N). In the initial stage (N < 250), Γ fluctuates violently, reflecting the disturbance of small-scale network structure adjustment to toughness. In the middle stage (250 ≤ N < 500), the fluctuation amplitude narrows, and Γ decreases continuously along the fitting curve. In the early warning stage (N = 500), Γ decreases to ≈0.01, and the system enters a high-risk early warning state.

The model predicts that the collapse point of the system is N = 736 and, at this time, the network completely loses its ability to resist risks, and the trust structure will fail as a whole.

Trust decay risk R increases quadratically with the fitting model R = 6.65N^2^ − 61.54N + 204.26. In the gradual accumulation period (N < 300), R remains low (≈0~80), and risk accumulates slowly. In the accelerated outbreak period (N ≥ 300), R rises rapidly and sharply to ≈900 at N = 500, and risk releases exponentially. N = 500 is the risk acceleration turning point. At this time, the risk growth rate increases significantly, which completely coincides with the resilience warning scale, marking that the system enters an irreversible decline–collapse channel. Resilience index is negatively coupled with trust risk. Scale expansion continues to weaken system resilience, while amplifying risk exposure, forming an evolution path of “resilience attenuation risk accumulation-accelerated explosion”. Scale control strictly limits the network size to N < 500 to avoid entering the risk acceleration zone. the early warning mechanism, with N = 500 as the threshold, constructs a “scale-resilience-risk” linkage early warning system to intervene in advance to prevent systemic collapse. Model value, exponential decay and the quadratic growth model provide a quantifiable prediction framework for risk management of large-scale trust networks.

This study is an extension of N = 10, 100 small-scale network analysis. Resilience fluctuations and risk germination observed in small-scale networks evolve into systematic decay and explosive growth in large-scale networks. Structural disturbances at the micro level eventually converge into trust collapse risks at the macro level, verifying the scale consistency of evolution laws.

### 5.4. Parameter Sensitivity Analysis of Trust Decay Risk

**Figure 12:**
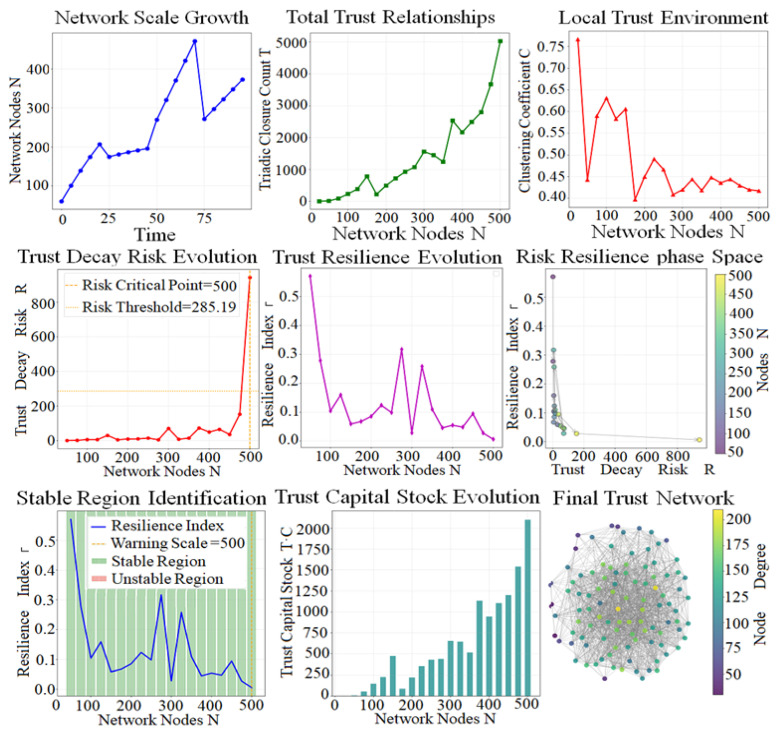
Comparative Analysis Comprehensive Diagram.

Based on the trust network model with linear expansion from 100 to 500 nodes, this study systematically analyzes the dynamic evolution law of trust systems in the process of scale growth from the dimensions of network structure, trust relationship, risk resilience and capital stock, providing a quantitative basis for scale control and risk governance of social trust networks and analyzing the parameter sensitivity of trust decay risk (as shown in Figure 12).

Figure 12 shows that the number of network nodes N fluctuates with time, providing a basic scale variable for the evolution of trust systems. The triadic closure number T (trust relationship density) fluctuates slowly when N < 350 and exponentially rises to 5000 after N > 350, indicating that local trust connections in large-scale networks close rapidly, forming high-density trust clusters.

Clustering coefficient C fluctuates violently at the beginning (N = [50, 100]) and converges gradually to ≈0.42, indicating that the local trust structure changes from unstable to equilibrium after network expansion, and the small-world characteristics are gradually prominent, which indicates that the trust environment tends to stabilize.

Trust decay risk is at a low level for a long time, far below the critical threshold of 285.19, but it breaks through the threshold instantaneously at N = 500, marking the outbreak point of systemic risk.

The overall fluctuation of the trust resilience index Γ decreases from 0.75 to 0.02 at N = 500, indicating that the network expansion continues to weaken the risk resistance, and significant resilience valleys appear at N = 150,300 and other nodes.

The phase space distribution of risk and resilience shows that small networks (N < 250) concentrate in the “low risk–high resilience” region, large networks (N > 250) concentrate in the “high risk–low resilience” region, and N = 500 is the most vulnerable state of the system.

Trust capital stock T*C increases rapidly with scale growth, at N = 500 reaching a peak value of ≈2000, consistent with the trend of total trust relationship T, indicating that trust assets are mainly driven by the number of connections. The stable region defined by resilience threshold of 500 covers most intervals of N = [50, 500], only at a few nodes (N = 50, 250, 300) showing transient instability, which indicates that the trust network has a good risk-resistant foundation at a moderate scale, which indicates that trust capital is basically stable in accelerating accumulation although a few nodes are unstable in the interval [50, 500].

When N = 500, a core–edge small-world network is formed, characterized by the coexistence of high-degree nodes (degree ≈ 200) in the core and low-degree nodes (degree ≈ 50) in the edge. High connectivity improves trust efficiency and provides a channel for risk propagation.

Network expansion significantly increases trust capital and connection density but simultaneously weakens system resilience and finally triggers systemic risk at N = 500, showing a scale-driven double-edged sword effect.

N = 500 is the critical risk scale of the trust network, and blind expansion to this node needs to be avoided. The network scale should be controlled at [250, 400] to balance trust accumulation and risk resistance. For node numbers with low resilience such as N = 150, 300, strengthen local connections and decentralize core power to improve risk resistance. Establish a trust decay risk threshold monitoring mechanism to intervene in advance to prevent systemic collapse.

This study quantitatively reveals the coupling evolution law of trust network scale, structure, risk and resilience and provides a reusable analytical framework and empirical basis for trust design and risk governance in social networks, organizational management, distributed systems and other fields.

## 6. Conclusions and Prospects

### 6.1. Main Conclusions

This study focuses on the central role of triadic closure structure in social media trust decay prevention, and the specific conclusions are as follows.

(1) Dynamic evolution of triadic closure is the key to trust anti-decay. The stability of clustering coefficient C, the short range of average path length L, the capacity of triadic closure number T and its growth rate constitute the structural index system of trust anti-decay. The forest fire model is proved to be an ideal model for simulating trust anti-decay networks because of its high clustering, short path and triadic closure power-law growth. Its structural characteristics are significantly better than those of ER and BA models.
(2) The risk of trust decay can be quantified. The risk index of trust decay proposed in this paper can effectively describe the change of structural vulnerability in the process of network growth. Distance correlation analysis reveals the specificity of risk evolution of different network models. The forest fire model has a significant risk peak at N ≈ 40 and the T-C relationship is not close, indicating the suddenness of trust decay and the necessity of monitoring and early warning. The BA model showed T-C negative correlation, and centralizing structure suppressed risk but sacrificed local clustering; the ER model had the lowest risk and T-C positive correlation, and the clustering coefficient could be used as a stability index.
(3) The trust resilience index defined in this paper verifies the inherent differences in anti-decay abilities of different networks, so that it can be used to predict theoretically when evaluating the trust stability of networks.
(4) For the structural index correlation model, we propose prevention mechanisms, including structural strengthening, dynamic compensation, path optimization, monitoring and early warning. For the forest fire network, we need to strengthen monitoring and active intervention at a critical scale; for the ER model network, we can set the clustering coefficient threshold to warn and compensate for connection; for the BA model network, we should restrain excessive growth of hub nodes and monitor their behavior; for complete graphs, we need to control their scale.

In a word, this research system reveals the core mechanism and quantitative law of triadic closure structure in preventing trust decay in social media and lays a theoretical foundation for building a dynamic, intelligent and structure-sensitive trust guarantee system.

### 6.2. Research Limitations and Prospects

This study has made progress in theoretical modeling and simulation analysis, but there are still some limitations, which also reveal the future direction worthy of further exploration.

Firstly, this model focuses on the evolution of macro network structure and fails to integrate the dynamic changes of micro user behavior (such as information sharing, blocking, reporting) and subjective trust perception. An important direction for future research is to combine computational sociology methods to build an agent-based model that integrates user cognition, emotion and behavior rules or to utilize fine-grained user interaction time series data to realize mechanism simulation and prediction of trust establishment, maintenance and decay processes at a more microscopic level.

Secondly, the structural index system and risk model proposed in this study need to be further studied in terms of computational efficiency and deployability in large-scale real-time systems. How to design a lightweight flow algorithm for approximate calculation and tracking of triangular closure number and clustering coefficient is an engineering challenge with practical significance.

Finally, the validation of this study is primarily based on platform-specific attention networks. Future work can be validated in different types of social networks (such as two-way friend networks, content interaction networks) and cross-platform data to test the universality of this framework and explore the role of cultural or platform characteristics in the evolution of trust structures.

## Figures and Tables

**Table 1:** Distance Correlation Analysis Table.

Type of Network	N-T Correlation	T-C Correlation	Attenuation Risk Model
empathetic group	0.92 **	0.85 **	R = 0.15N^2^ − 1.2N + 8.3
ER random graph model	0.78 **	0.62 *	R = 0.08N^2^ − 0.7N + 5.1

** *p* < 0.01, * *p* < 0.05.

**Table 2:** Examples of Early Warning Response for Different Network Types.

Type of Network	Newly Added Node	Risk Changes	Warning Level	Prevention Strategy
forest fire	5	0.15	high risk	activate 3-hop validation and behavioral analysis
BA model	10	0.02	medium risk	limit new connections
ER random graph	20	0.03	medium risk	increase cluster monitoring frequency

## Data Availability

The raw data supporting the conclusions of this article will be made available by the authors on request.

## Abbreviations

The following abbreviations are used in this manuscript:

ER	Erdős–Rényi Model
BA	Barabási–Albert Model
